# Maternal diet, aging and diabetes meet at a chromatin loop

**DOI:** 10.18632/aging.100330

**Published:** 2011-05-25

**Authors:** Susan E. Ozanne, Ionel Sandovici, Miguel Constância

**Affiliations:** ^1^ Metabolic Research Laboratories, Institute of Metabolic Science, University of Cambridge, Cambridge CB2 OQQ, United Kingdom; ^2^ National Institute for Health Research Cambridge Biomedical Research Centre, Cambridge, United Kingdom; ^3^ Metabolic Research Laboratories, Department of Obstetrics and Gynaecology University of Cambridge, Cambridge CB2 0SW, United Kingdom; ^4^ Centre for Trophoblast Research, University of Cambridge, Cambridge CB2 3EG, United Kingdom

**Keywords:** nutrition, aging, epigenetic, Hnf4a, type 2 diabetes

## Abstract

We have recently demonstrated that exposure to a suboptimal diet during early development leads to abnormal epigenetic regulation of a promoter-enhancer interaction at the gene encoding HNF-4α, a key transcription factor required for pancreatic β-cell differentiation and glucose homeostasis. In addition, our studies revealed that the suboptimal maternal diet amplifies the age-associated epigenetic silencing of this locus. In this research perspective we discuss these novel findings in the context of the growing list of epigenetic mechanisms by which the environment can affect gene activity and emphasize their implications for the understanding of the mechanistic basis of the development of type 2 diabetes with age.

## Maternal diet and the developmental origins of type 2 diabetes

It is well established that what we eat has a major impact on our health. However, there is growing evidence to suggest that diet during pregnancy and lactation may be particularly important as not only does it influence the health of the mother, it may have a permanent effect on the health of her children and even her grandchildren. The concept that environmental factors, such as nutrition during early development, influence both our health span and lifespan has been termed the developmental origins of health and disease hypothesis [1]. Focus on this possibility was prompted by the results of a series of epidemiological studies that revealed relationships between patterns of early growth and long-term risk of metabolic conditions such as type 2 diabetes (T2D) and cardiovascular disease [1]. Initial studies demonstrated that there was a linear relationship between birth weight and prevalence of these conditions in later life, with individuals with the lowest birth weight being around six times more likely to have T2D than those with the highest birth weight [2]. These associations have been replicated in numerous populations worldwide representing multiple ethnic groups, though those in some of the more contemporary cohorts where there was a high prevalence of maternal obesity also revealed an increased risk of metabolic disease in the very high birth weight offspring [3]. Subsequent studies demonstrated that the detrimental effects of being born small for gestational age were exaggerated if an individual grew rapidly in early postnatal life [4]. It was also demonstrated that accelerated postnatal growth, independently of growth *in utero*, increased the risk of metabolic conditions such as obesity [5].

Observational studies linking patterns of early growth with long-term health don't provide proof that suboptimal nutrition mediates these relationships. However studies in human cohorts and in animal models have provided strong evidence that environmental factors, including nutrition, play an important role. The strongest evidence from human studies supporting the role of the environment in mediating the relationships between birth weight and long-term health has come from the study of monozygotic (identical) twins. These studies revealed that in monozygotic twin pairs discordant for T2D, the diabetic twin has a lower birth weight than their non-diabetic co-twin [6,7] Assessing the role of maternal diet in mediating the relationships between fetal growth and long-term health in a human context is complex. However, studies of individuals *in utero* during periods of famine have shown direct relationships between severe maternal nutrient deficiency and increased risk of T2D in the offspring [8,9]. The importance of neonatal nutrition for long-term health has been demonstrated very clearly in human studies. Initial observational studies demonstrated the protective effects of breast-feeding on future risk of metabolic disease [10] and subsequent randomized intervention studies revealed causal relationships between nutrition during early postnatal life and long-term metabolic health [11]. Animal models have provided compelling evidence that maternal diet during pregnancy and lactation influence long-term health including risk of T2D, obesity, hypertension and cardiovascular disease. The developmental origins of health and metabolic disease is therefore widely accepted.

## Interaction between maternal diet and aging in T2D risk

Most conditions associated with patterns of early growth and nutrition are diseases associated with aging. Therefore, perhaps not too surprisingly, the small size at birth has been associated with increased mortality in humans. In a large Finnish study a low birth weight was associated with increased mortality at all ages in women and with premature death (< age 55) in men [12]. Studies in rodent models have also shown direct associations between maternal diet and lifespan of the offspring [13,14]. Maternal protein restriction during pregnancy resulting in low birth weight, followed by postnatal catch-up growth through suckling by normally fed dams (recuperated offspring) led to a reduction in lifespan. In contrast, maternal protein restriction and slow growth during the lactation period resulted in increased life span and conferred protection from the detrimental effects of an obesogenic diet. In addition to differences in aging at the whole body level, these same rodent models have revealed effects of maternal diet on aging at the cellular level, as demonstrated by differences in rates of telomere shortening and in markers of cellular senescence [15].

The islets of Langerhans in the endocrine pancreas, the only cells in the body that can produce insulin, appear to be particular vulnerable to the detrimental effects of maternal diet on their aging trajectory. This is consistent with the early environment having a major influence on regulation of glucose homeostasis and consequently diabetes risk. Some of the earliest effects on telomere shortening resulting from maternal protein restriction are observed in pancreatic islets and this is accompanied by premature induction of p16, one of the most robust biomarkers of aging [15]. In addition, maternal protein restriction during pregnancy and lactation results in increased age-associated oxidative stress and development of fibrosis [16].

**Figure 1.**
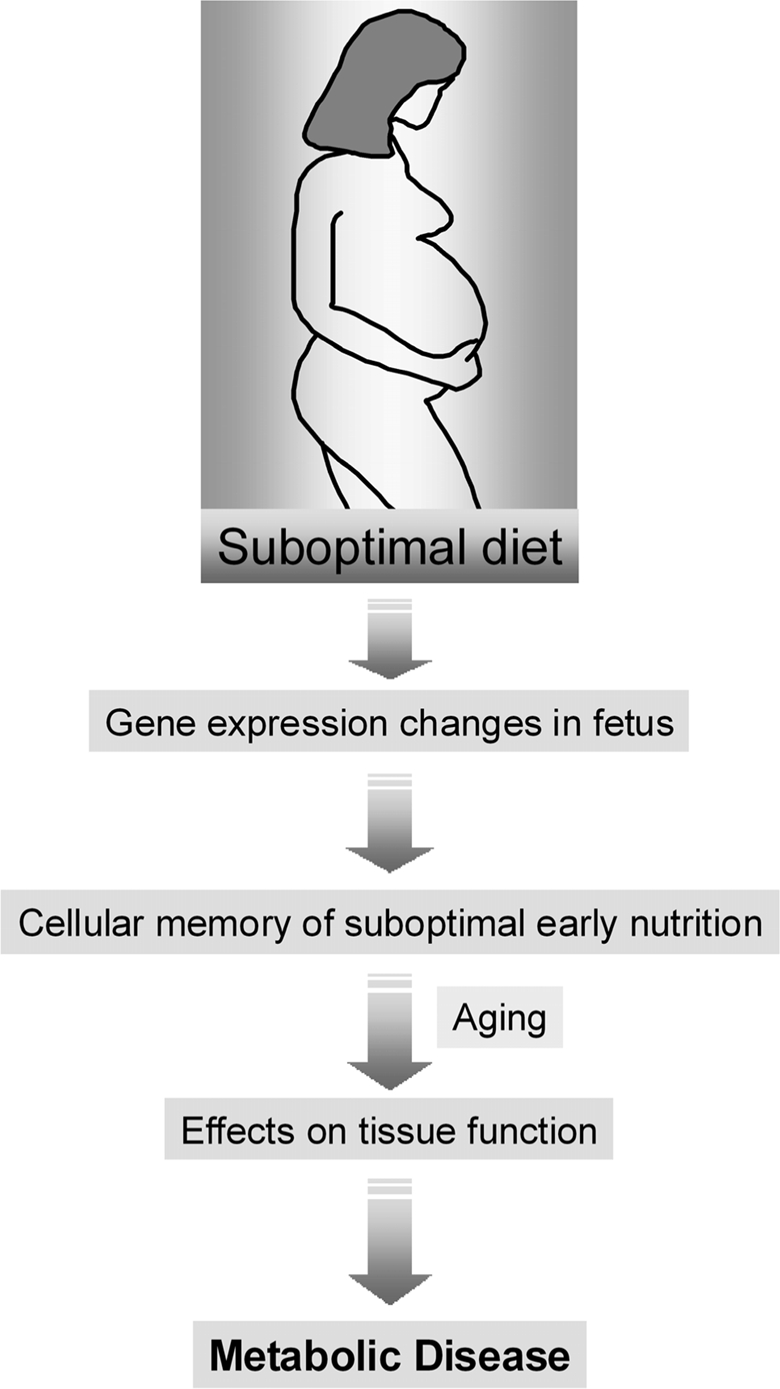
**The role of maternal diet in the development-tal origins of metabolic disease.** An abnormal diet during pregnancy can induce gene expression changes in the developing fetus. These changes are then perpetuated during many subsequent rounds of cell division throughout the lifetime of the offspring (cell memory) and may lead to an increased disease risk in the adulthood.

The molecular bases of the interaction between maternal diet, aging and the risk for T2D are currently poorly understood. However, the hypothesis that this interaction could be mediated by epigenetic mechanisms offers an attractive explanation for the link between nutrition, regulation of gene expression, and the risk for disease (Figure 1).

## Epigenetic dynamics during early development and aging

Gene transcription is the result of the interactions between transcription factors and chromatin at a number of genomic regulatory elements including promoters, enhancers, insulators and silencers. Accessibility to these sites is mediated by epigenetic modifications of histones and DNA. It is widely accepted, for example, that upon histone acetylation, genes become actively transcribed, whereas deacetylation leads to repression. Modifications of the DNA itself, such as methylation and most recently hydroxymethylation, are also important indicators of the transcriptional regulatory activity of many genomic loci.

Epigenetic marks are responsive to environmental and developmental cues, as there is a need to modify the transcriptional outputs of genes throughout the life of an organism [17,18]. It is clear that chromatin structure changes in response to the cell's many stimuli. Certain epigenetic marks can be added and removed before a cell divides or within few cell divisions. Such short-term flexibility is particularly important to allow appropriate responses to acute environmental cues. Other epigenetic marks can be maintained for many cell divisions. Long-term stability is required for epigenetic programming of the early embryo, a process that refers to the acquisition and stable propagation of marks that define cell types and maintain cellular memory [18].

Deregulation of epigenetic processes with age is a common feature in mammals [19]. Widespread and tissue-specific age-related DNA methylation changes have been reported in many mouse and human studies [20]. It is also becoming increasingly clear that diseases associated with aging, such as T2D, may have an important epigenetic component [18,21]. However, little is known about the interactions between environmental exposures, aging and epigenetic variation that may lead to the increased disease risk.

## Regulation of promoter-enhancer interactions as a novel epigenetic mechanism by which maternal diet and aging influence long-term health

A recent study in rats sheds novel insights on the epigenetic mechanisms by which environmental exposures can increase the risk of T2D [22]. Sandovici and co-workers used a well-established model for the developmental origins of T2D to study the interaction between early life sub-optimal nutrition, aging and epigenetic states at a locus encoding a key transcription factor: HNF4-α. Rats exposed to a low protein (LP) maternal diet during gestation and lactation showed reduced *Hnf4a* transcription at a young age (3 months, 3M) that persisted throughout life when compared to normally fed controls. LP offspring develop T2D at old age (17M), whereas normally fed controls rarely do so. The authors showed that the reduced *Hnf4a* expression observed at a young age was made permanent throughout life and aging, via the stable maintenance of repressive epigenetic marks. Remarkably, the early-diet epigenetic silencing effects were targeted to a regulatory element distal to the active promoter - an enhancer element. The abnormal epigenetic marking weakened the physical interaction with the upstream promoter and resulted in reduced expression of the gene. Interestingly, the accumulation of the repressive marks were more pronounced in aged rats that were exposed to a sub-optimal diet in early life than those exposed to a normal diet, which is suggestive of an interaction between early-nutrition and aging effects at this locus. Down-regulation of *Hnf4a* has been found in T2D patients [23] and genetic polymorphisms within or nearby this gene are associated with T2D risk [24]. The sustained lifelong reduction of *Hnf4a* resulting from de-regulation of an epigenetically controlled enhancer-promoter interaction is thus likely to contribute to the T2D effects in this model. This work is relevant to human T2D risk, as the epigenetic mechanisms that regulate *Hnf4a* in rats are highly conserved in human islets [22].

**Figure 2.**
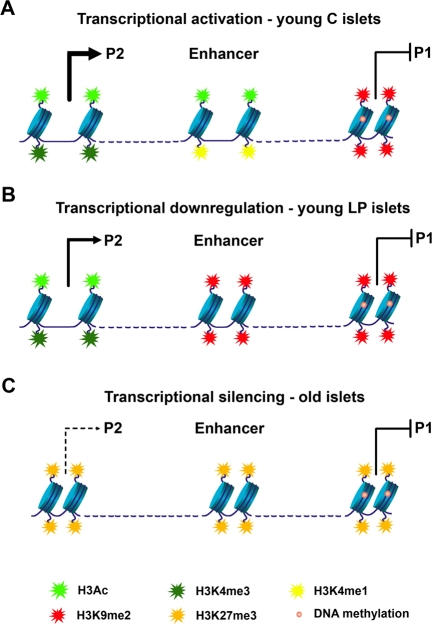
**Model of epigenetic control at the *Hnf4a* locus.** (**A**) In young control islets the P2 promoter has low levels of DNA methylation and is associated with open chromatin and high levels of transcription (enrichment in H3Ac and H3K4me3). An active downstream enhancer (enriched in H3Ac and H3K4me1) is essential for high levels of P2 transcription. Accessibility of transcription factors to the P1 promoter is blocked by DNA methylation and H3K9me2. (**B**) Down-regulation of P2 transcriptional activity in young LP islets results from loss of enhancer activity. The enhancer adopts a closed chromatin configuration with enrichment in H3K9me2 and depletion of active histone marks. (**C**) Transcriptional silencing along the *Hnf4a* locus during aging is achieved especially by recruitment of H3K27me3.

Several HNF-4α protein variants can arise from mRNAs transcribed from two promoters, proximal P1 and distal P2, however, in islets only the P2 promoter is active. An enhancer region is located upstream of the P1 promoter and downstream of the P2 promoter (Figure 2A). This shared enhancer increases the activity of both promoters in a tissue-specific manner. In islets, the chromatin configuration of the P1 promoter differs dramatically from that of the P2 promoter and the enhancer region. Accordingly, P1 is enriched in histone repressive marks and has high levels of DNA methylation, leading to closed chromatin, whereas the P2 promoter and the enhancer are characterized by open chromatin (Figure 2A). This epigenetic pattern dictates the activity of the promoters, with P2 being the active one in islets (Figure 2A). The effects of early life exposure to a maternal LP diet were specific to the enhancer region - with recruitment of the repressive mark H3K9me2 and loss of a mark predictive of active enhancers, H3K4me1 (Figure 2B). In turn, aging led to widespread epigenetic silencing across the locus (Figure 2C). Most prominently, the Polycomb-dependent histone repressive mark H3K27me3 was found enriched at the P2 and the enhancer regions with age. In this study, causality effects of the epigenetic marking on transcription levels were inferred through pharmacological inhibition of specific histone modifications and of DNA methylation in insulin-secreting cell lines.

The work by Sandovici *et al*. provides novel insights into the epigenetic deregulation during aging and the associated decline in function of the endocrine pancreas. *Hnf4a* levels decline with age, an effect that was associated with recruitment of the Polycomb-mediated histone repressive mark H3K27me3. Repression by H3K27me3 across the P2 and enhancer regions was greater in magnitude in rats fed a sub-optimal LP maternal diet compared to controls. This finding raises several questions: does age-related H3K27me3 repression occur earlier in LP rats compared to controls, which would be indicative of premature epigenetic “aging” at this locus? Do H3K27me3 levels contribute to the age-associated increased risk for diabetes? (LP rats, with higher enrichment of H3K27me3 develop diabetes, whereas control rats with lower levels of H3K27me3 are diabetes-free) Is age-related Polycomb-mediated accumulation of H3K27me3 associated with repression of other key diabetes genes in islets?

Prior to the work by Sandovici *et al*. there was only one published report analysing the effects of early life-events on epigenetic states during aging [25]. In this study, a daily 3 hour separation of mouse pups from their mother during postnatal days 1-10 caused persistent hypomethylation at specific CpG sites of the arginine vasopressin (*Avp*) gene enhancer at 6 weeks of age, with age-associated hypomethylation (at 1 year of age) occurring stochastically across the entire *Avp* locus [25]. The study by Sandovici *et al*. similarly shows that early-life epigenetic changes are targeted towards important regulatory elements, while age-associated effects are widespread and likely stochastic. However, in contrast to the *Avp* study, the differential changes in epigenetic states at the *Hnf4a* locus in response to early-life events and aging seem to be mechanistically and functionally linked. Indeed, whilst the age-associated hypomethylation of the *Avp* had no effect on mRNA expression levels, *Hnf4a* repression by histone modifications with age resulted in lower levels of expression. Furthermore, the early-diet induced epigenetic programming effects at the enhancer were largely maintained during aging, and were even more pronounced for the Polycomb-dependent histone mark H3K27me3. Therefore, the aging process did not disrupt the long-lasting programming effects at this locus and may have instead amplified its effects.

**Figure 3.**
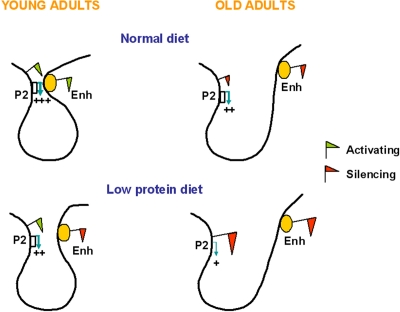
**Proposed simplified model for the enhancer-promoter looping at the *Hnf4a* locus in young and old C and LP islets.** In islets from young adult C rats the enhancer is enriched in active histone marks and interacts closely with the *Hnf4a* P2 promoter, leading to high levels of P2 transcription. The P2 promoter-enhancer interaction is impaired in islets of animals exposed to a LP diet during early development, due to depletion of active histone marks and enrichment in suppressive histone marks at the enhancer region. Aging associates reduced levels of *Hnf4a* expression due to enrichment in suppressive histone marks across the locus, which is more pronounced in LP islets. The accumulation of suppressive histone marks might further weaken the P2 promoter-enhancer interaction.

The work by Sandovici *et al*. showed that environmental stimuli could induce specific changes in epigenetic states at an enhancer region and provided insights into the mechanisms by which transcript levels were altered. By using the chromatin conformation capture (3C) technology the study identified a stable P2 promoter-enhancer interaction in islets that determines the establishment of a functional expression domain (Figure 3). This interaction was much weaker in islets isolated from LP offspring (Figure 2) and in insulin-secreting cell lines, both of which have low levels of P2 transcripts, as well as in liver, a tissue that does not expresses P2 transcripts. The strength of the promoter- enhancer interaction is likely to be primarily mediated by the epigenetic state of the enhancer, i.e. a closed configuration by recruitment of repressive marks leads to a weaker interaction (Figure 3). If this is true, one could extrapolate that the P2 promoter-enhancer interaction in islets will be weaker with aging (Figure 3), although this hypothesis has not been experimentally tested. On the basis of this model Sandovici *et al*. proposed that the epigenetic control of the P2-enhancer interactions in islets underpins the sustained changes in *Hnf4a* expression levels, which influence in turn the risk for the age-associated T2D. It is tempting to speculate that this model could also provide a mechanistic understanding of how polymorphisms within the human *Hnf4a* locus may increase T2D risk, as the genetic sequence, in addition to the epigenotype, could modulate the strength of the promoter-enhancer interaction.

## Perspective

The findings by Sandovici *et al*. led to the proposal that altered epigenetic control of enhancer-promoter interactions are crucial for establishing long-lasting programming and aging effects. The catalogue of environmentally-induced epigenetic modifications that affect enhancers is still rather limited, including so far the *Avp* enhancer mentioned above [25], *Ppara* [26] and now *Hnf4a* [22]. Using genome-wide unbiased approaches to identify cell-type specific chromatin signatures at enhancer regions during early-life programming and aging has the potential to define many more. Indeed, efforts to identify regulatory elements, i.e. enhancers, repressors and insulators, are now well underway taking advantage of the advancement of genome-wide approaches [27-29]. One such study performed in human cell lines suggests the existence of a vast number of enhancers in the genome, in the order of 10^5^-10^6^, that are used to drive specific gene expression patterns in the estimated 200 cell types of the human body [28]. Results from this study suggest that enhancers are the most variable class of transcriptional regulatory elements between cell types in terms of epigenetic signatures. On the contrary, the chromatin states at promoters and the CTCF binding at insulators were found to be largely invariant across diverse cell types [28]. The effects of early-life adverse effects and aging on enhancer-promoter interactions and, more widely, on long-range genomic interactions [30,31] is a promising area of future research that may add important insights to our understanding of the underlying mechanisms of age-associated diseases, and that may lead eventually to the discovery of novel treatments.
